# Supplementation of Re-Esteriﬁed Docosahexaenoic and Eicosapentaenoic Acids Reduce Inflammatory and Muscle Damage Markers after Exercise in Endurance Athletes: A Randomized, Controlled Crossover Trial

**DOI:** 10.3390/nu12030719

**Published:** 2020-03-09

**Authors:** Domingo J. Ramos-Campo, Vicente Ávila-Gandía, Fco Javier López-Román, José Miñarro, Carlos Contreras, Fulgencio Soto-Méndez, Joan C. Domingo Pedrol, Antonio J. Luque-Rubia

**Affiliations:** 1 Faculty of Sports, UCAM, Catholic University San Antonio, 30107 Murcia, Spain; djramos@ucam.edu; 2 Department of Exercise Physiology, Catholic University San Antonio, 30107 Murcia, Spainjmm@mozosdelconvento.com (J.M.); cjcontreras@ucam.edu (C.C.); fsoto@ucam.edu (F.S.-M.); ajluque@ucam.edu (A.J.L.-R.); 3 Department of Biochemistry and Molecular Biology, School of Biology, University of Barcelona, 08028 Barcelona, Spain; jcdomingo@ub.edu

**Keywords:** creatine kinase, IL-6, omega-3 fat, resistance exercise, strength

## Abstract

This study aimed to analyse the effect of 10 weeks of a highly concentrated docosahexaenoic acid (DHA) + eicosapentaenoic (EPA) supplementation (ratio 8:1) on strength deficit and inflammatory and muscle damage markers in athletes. Fifteen endurance athletes participated in the study. In a randomized, double-blinded cross-over controlled design, the athletes were supplemented with a re-esterified triglyceride containing 2.1 g/day of DHA + 240 mg/day of EPA or placebo for 10 weeks. After a 4-week wash out period, participants were supplemented with the opposite treatment. Before and after each supplementation period, participants performed one eccentric-induced muscle damage exercise training session (ECC). Before, post-exercise min and 24 and 48 h after exercise, muscle soreness, knee isokinetic strength and muscle damage and inflammatory markers were tested. No significant differences in strength deficit variables were found between the two conditions in any of the testing sessions. However, a significant effect was observed in IL1β (*p* *=* 0.011) and IL6 (*p* *=* 0.009), which showed significantly lower values after DHA consumption than after placebo ingestion. Moreover, a significant main effect was observed in CPK (*p =* 0.014) and LDH-5 (*p =* 0.05), in which significantly lower values were found after DHA + EPA consumption. In addition, there was a significant effect on muscle soreness (*p =* 0.049), lower values being obtained after DHA + EPA consumption. Ten weeks of re-esterified DHA + EPA promoted lower concentrations of inflammation and muscle damage markers and decreased muscle soreness but did not improve the strength deficit after an ECC in endurance athletes.

## 1. Introduction

Long-chain omega-3 polyunsaturated fatty acids (PUFAs), which are mainly contained in fish oil, continue to receive research attention. Namely, eicosapentaenoic acid (EPA; 20:5 *n*-3) and docosahexaenoic acid (DHA; 22:6 *n*-3) compete with arachidonic acid for incorporation into phospholipidic cell membranes, helping to decrease chronic inflammation by reducing the activation of the nuclear factor kappa-light-chain-enhancer of activated B cells (NF-KB) at the genomic level [1,2] and acting acutely through inflammation mediators like prostaglandins, leukotrienes, lipoxins, resolvins and protectins [3]. These PUFAs can undergo enzymatic reactions that produce lipid mediators, as well as interacting with oxygen free radicals [4]. It has been demonstrated that pleiotropic properties of the peroxidation products of this PUFA affect both oxidative and anti-oxidant response pathways [5]. Thus, it is widely known that PUFAs are an effective nutritional aid for improving health markers [6]. For example, PUFAs reduce blood pressure [7], improve cardiovascular function [8], enhance cognitive function [9] and decrease depression symptoms [10].

On the other hand, scientists and coaches are continually looking for techniques to improve exercise performance [11], the intake of ergogenic aids being one of the most common and popular means used by athletes to maximise their physical performance [12]. Specifically, exhaustive exercise (e.g., high-intensity exercise, eccentric exercise or resistance training) produces muscular myofibril ruptures causing inflammation, muscle damage with a concomitant increment of the plasma concentrations of muscle damage markers, delayed onset muscle soreness (DOMS) and muscle fatigue, decreasing sports performance [11]. In this way, there is some evidence that *n*-3 PUFA supplementation has a positive ergogenic effect, improving exercise performance and recovery processes [13,14] and contributes to reducing exercise fatigue [6]. In addition, PUFAs ingestion improves antioxidant, muscle damage and anti-inflammatory responses [15] and reduces delayed-onset muscle soreness [6] after DHA supplementation, leading to a higher anti-inflammatory potential than other *n*-3 PUFAs such as the EPA [16]. In addition, many studies have reported a decrement of DOMS after EPA and DHA intake [17,18]. However, other studies have found no effect on DOMS [15,19] and muscle damage markers [19] after 2 weeks of 0.8 g/day of DHA intake. Therefore, the findings are controversial. Therefore, the anti-inflammatory potential and muscle soreness reduction capability of PUFAs has increased research attention to PUFAs as a potential ergogenic aid in the context of enhancing sport performance.

On the other hand, the effect of PUFAs intake on strength performance remains unclear because the results of studies which have analysed the response to this fatty acid are controversial. Some studies [17,18] have observed that ingestion of EPA and DHA causes an inhibition in torque deficit (17%), but other studies [20,21] have found no ergogenic effect of EPA + DHA or only DHA [15] supplementation on strength performance. However, some studies [6] have reported that long periods of supplementation (over an 8-week period) are necessary to achieve the ergogenic effect of EPA and DHA intake on strength performance.

Interestingly, previous studies which analysed the effect of PUFAs on strength performance and the inflammatory and muscle damage response after eccentric exercise used untrained participants. However, this fatty acid would be an especially interesting aid in sports modalities (i.e., endurance athletes) which need to develop several capacities simultaneously due to their faster time course of recovery [22]. Thus, taken together, the current findings and those of previous studies support the idea of DHA + EPA intake during long periods of supplementation to obtain an effective ergogenic effect on inflammation, muscle damage markers and strength performance. Therefore, the aim of this investigation was to analyse the ergogenic effect of 10 weeks of highly concentrated DHA + EPA supplementation (DHA:EPA ratio equal to approximately 8:1) on strength performance and inflammatory and muscle damage markers in endurance athletes. The initial hypothesis was that DHA + EPA supplementation would achieve less muscle soreness, lower values of inflammation and muscle damage markers and a lower decrement in strength parameters than the placebo.

## 2. Materials and Methods

### 2.1. Design

**Figure 1:**
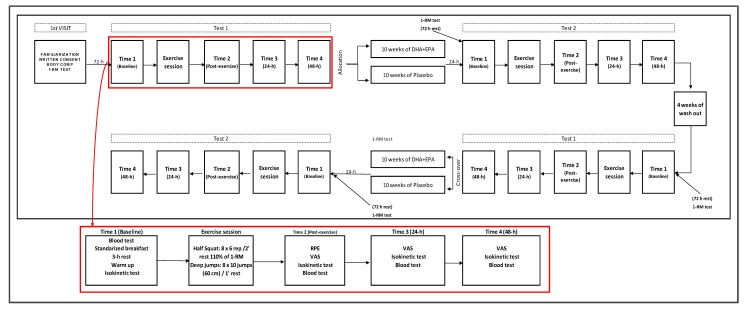
Schematic explanation of the protocol applied in the study.

A randomized, double-blinded cross-over controlled design was employed to analyse the effect of 10 weeks of DHA + EPA acids supplementation or placebo (PLA) on strength performance and inflammatory and muscle damage markers. The participants were supplemented in randomized order with 10 weeks of DHA + EPA or PLA, and after a 4-week wash out period, athletes were exposed to the other experimental condition (DHA + EPA or PLA). The trial followed the CONSORT guidelines, and it was registered with ClinicalTrials.gov (Ref. NCT04125199). Randomization was performed using software (Epidat 4.2, 2016) which generated random codes assigned to participants. An initial testing session composed of an exercise session and four measurement time points (baseline, post-exercise, 24 h and 48 h post-exercise) was carried out. Afterwards, supplementation was conducted for 10 weeks subsequently performing a second testing session with the same characteristics. After a wash out period, the same procedure was applied, but athletes were exposed to the other experimental condition (Figure 1).

### 2.2. Participants

**Figure 2:**
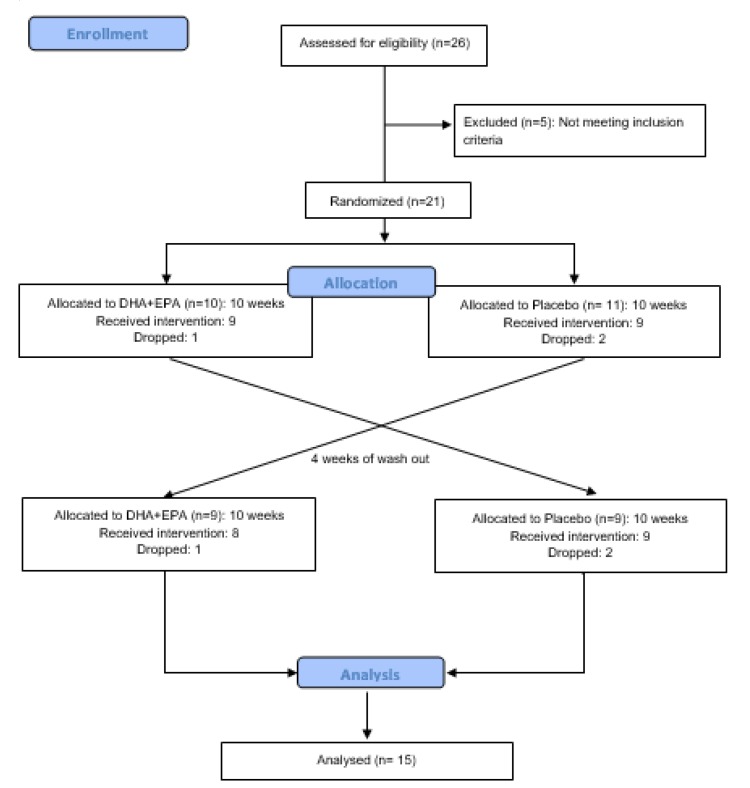
Flow diagram of the progress of the randomized trial.

Twenty-six healthy male amateur endurance athletes were invited to participate in the study but five did not meet the following inclusion criteria: (a) aged 18–45 years; (b) at least 2 years of endurance training experience including strength training in their training routine; (c) at least 3 days per week of training; (d) normal BMI (19–25 kg·m^-2^). The exclusion criteria were (a) chronic pathologies; (b) alcohol drinkers or smokers; (c) intake any nonsteroidal anti-inflammatory drug or supplement during the study; (d) diets with an excess of antioxidant or anti-inflammatory products; (e) injury or musculoskeletal disorder during the three months prior to the study. While twenty-one participants were originally recruited to participate in this study, only fifteen participants were included for analysis (age: 36.0 ± 8.1 years; height: 179.3 ± 7.3 cm; weight: 80.4 ± 11.2 kg; fat mass: 10.2 ± 4.8%; training experience: 6.4 ± 3.1 years; VO_2_max: 61.9 ± 3.1 ml/kg/min). Six participants were unable to complete the study protocol due to injury (n = 2), unresolvable scheduling conflicts (n = 2) and compliance with supplementation (n = 2). All participants were instructed to maintain their regular dietary consumption during the study and to avoid ingesting caffeine or alcohol for at least 24 h before each visit. A written consent was obtained from each volunteer in accordance with the Declaration of Helsinki. In addition, the study was approved by the UCAM ethics committee (Ref CE041811). The flow diagram of the process is shown in Figure 2.

### 2.3. Procedures

Before the start of the first training session, all the participants performed one session of familiarization. The aim of this period was to familiarize participants with the isokinetic test, methodology and characteristics of the exercise session and to ensure proper strength exercise technique. During the session of familiarization, body composition was assessed with a segmental multifrequency bioimpedance analyser Tanita BC-600 (Tanita Corp., Tokyo, Japan), which uses an eight-point tactile electrode method to take readings from the body and following previous recommendations [23]. In addition, the one repetition maximum (1-RM) loads of half-squat was determined as described elsewhere [24].

Three days later, participants started the first step of the first testing session. First, a haematological test was developed following previous procedures in this population [25]. The blood sample (10 ml) was withdrawn from an antecubital vein using a sterile technique to analyse haematological variables. Blood samples were taken before breakfast after an overnight fast. Blood extraction was performed with the subject was seated. The following variables were analysed: (a) muscle damage: lactate dehydrogenase (LDH; UI/l) (1–5) and creatine phosphokinase (CPK; UI/l); (b) inflammatory markers: Interleukin 1 beta (IL1β; pg/ml); interleukin 6 (IL6; pg/ml); interleukin 8 (IL8; pg/ml); C-reactive protein (C-RP; mg/dl) and tumour necrosis factor alpha (TNFα; pg/ml).

In addition, a nutritionist performed an initial prospective 24-h dietary recall to assess participants’ diets. Afterwards, a 7-day food record with qualitative and quantitative data along with a printed guide for proper filling was given to them to calculate daily average intake through the first week of the treatment, and calculated using software (Cronometer Software Inc., https://cronometer.com). This nutritional assessment was performed again at each baseline of each supplementation condition (DHA + EPA and placebo) and test (test 1 and 2).

After blood collection, participants ingested a standardized breakfast and rested for 3 h. Later, participants warmed up prior to testing by cycling for 5 minutes on a stationary bicycle, working at 100 W and 70–80 rpm and performed 5 minutes of dynamic stretching for the main locomotive lower limb muscles. After the warm-up, the isokinetic test was performed by using a dynamometer (Biodex Medical Systems 3, Shirley, NY, USA). The exercise protocol was undertaken from a seated position (120° hip angle) after the participants were stabilized according to the manufacturer’s instructions. Participants completed 2 sets of 30 repetitions of knee flexion and extension in the right leg at an angular velocity of 60°/s and 3 minutes of rest between sets. Parameters such as chair height, backrest distance, seat level and dynamometer base were adjusted for each subject.

### 2.4. Blood Collection, Handling, Storage and analysis

Blood was collected into EDTA plasma and serum vacutainers tubes. Plasma samples were immediately centrifuged, whilst serum samples were incubated at room temperature for 30 min before centrifugation at 4000 × *g* for 20 min at 4 °C. Separated plasma and serum were aliquoted and stored at −80 °C until biochemical assays were performed. Serum creatine phosphokinase, lactate dehydrogenase (1–5), and C-reactive protein were analyzed in duplicate using a clinical chemistry analyzer (BK400, Biobase, Jinan, China). Plasma levels of IL-1β, IL-6, IL-8 and TNF-α were measured through an antibody-linked, fluorescently labelled microsphere bead based multiplex analysis system (Linco Research/Millipore, Billerica, MA) and quantified by Luminex100ISv2 equipment. The intra and inter-assay coefficient of variation for each cytokine was IL-1β: 7% and 12%; IL-6: 2% and 10%; IL-8: 3% and 14% and TNFα: 3% and 19%, respectively.

Five minutes after the isokinetic test, participants started eccentric-induced muscle damage exercise training. The main objective of the exercise protocol was to induce inflammation, muscle damage and soreness during the following days after exercise. During the training session, athletes performed eight sets of six repetitions of half-squat at 110% of 1-RM with 2 minutes of rest between sets in a multipower machine (Technogym, Cesena, Italy). Participants only performed the eccentric phase, while two assistants raised the load to start the next repetition. Athletes were instructed to lower the load in 3 seconds. When athletes finished the task, ratings of perceived exertion (RPE) were determined using the Borg scale. Five minutes after the participants finished the last repetition of half-squat, they performed 8 sets of 10 repetitions of drop vertical jump using a 60 cm platform with a 1-minute rest between sets. Athletes were instructed to drop off the box and immediately perform a maximal vertical jump.

Five minutes after the exercise session finished, participants were tested again (time 2: post-exercise). Isokinetic tests and blood sample collections were carried out using the same procedures as at baseline. In addition, muscle soreness of the lower limbs was measured by using a 100-mm visual analogue scale (VAS).

The same three tests (isokinetic test, blood sample collection and VAS) were performed again by the participants 24-h (time 3) and 48-h (time 4) after the exercise session.

During the last evaluation (time 4), participants were allocated to one of the experimental conditions (DHA + EPA or PLA). They were supplemented for 10 weeks, and the same testing session, composed of an exercise session and four measurement time points (baseline, post-exercise, 24 h and 48 h post-exercise), was carried out. Finally, after a 4-week wash out, the athletes performed the other experimental condition (testing 1 followed by 10 weeks of supplementation and testing session 2). The 1-RM test was assessed again three days before each eccentric exercise training session.

### 2.5. Supplementation Protocol

Volunteers ingested six soft gels of DHA + EPA (Brudy Plus, Brudytechology, Barcelona, Spain) or PLA (500 mg of placebo of olive oil) provided by the same manufacturer, both products identical in appearance. The composition per soft-gel of DHA + EPA was: 0.45 mg of alpha tocopherol, 53.9 mg of glycerin and 139.1 mg of gelatin, 350 mg of DHA, 40 mg of EPA, total omega-3 content 405 mg and total fatty acid content 500 mg. The total daily dose was 2.1 g of DHA and 240 mg of EPA in 2.34 g of total omega-3 PUFAs consumed in a single dose in the morning before breakfast, for 10 weeks. Participants were asked to return the empty packs to ensure compliance.

### 2.6. Statistical Analysis

A data analysis was performed by using the statistical package SPSS v.24 (IBM, New York, USA). Descriptive statistics with measures of central tendency and dispersion were used. The assumption of normality and homoscedasticity was verified with the Shapiro–Wilk Test. A three-way analysis of variance (supplement (DHA + EPA and Placebo) × test (test 1 and test 2) × time (baseline, post-exercise, and 24-h and 48-h post-exercise) with repeated measures and a Bonferroni post-hoc test was used to investigate differences between study variables. For all the procedures, a level of significance of 0.05 was chosen.

## 3. Results

All subjects successfully finished the supplementation protocol. Compliance with supplementation during the intervention period was 99.4 ± 0.4%. The seven-day questionnaire showed that macronutrient values were similar between groups, for every nutritional variable (energy, protein, carbohydrates and total fat). Total fat breakdown also showed homogeneity (saturated, monounsaturated and polyunsaturated fat, further specified as omega-6, omega-3 and the omega-6/omega-3 ratio). Therefore, we assumed that dietary habits were preserved during the study.

Concerning inflammatory variables (Table 1), a significant effect (supplement × test × time) was observed in the IL1β (*F =* 4.247; *p =* 0.011) showing significantly lower values after DHA + EPA consumption than after placebo ingestion. In this way, compared with the first test, significantly lower values after DHA+EPA ingestion were shown post-exercise (*p =* 0.036), 24 h (*p =* 0.002) and 48 h *(p =* 0.004) after exercise.

**Figure 3:**
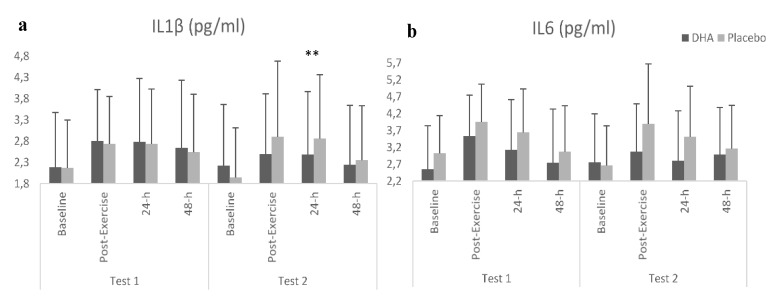
Results of inflammatory markers. **a**: IL1β; **b**: IL6. ** Differences between DHA + EPA and Placebo *p* < 0.01.

Moreover, when compared with the placebo, IL1β was significantly lower (*p =* 0.004) 24 h after exercise in the second test after DHA ingestion (Figure 3a). In addition, there was a significant effect on IL6 (*F =* 5.909; *p =* 0.009), with lower values for DHA + EPA compared with the placebo (Figure 3b). Thereby, compared with the first test, a statistical trend to obtain lower values after DHA + EPA ingestion were shown post-exercise (*p =* 0.062) and 24 h (*p =* 0.067) after exercise. However, no main effect was found on IL8, TNFα and C-RP.

**Figure 4:**
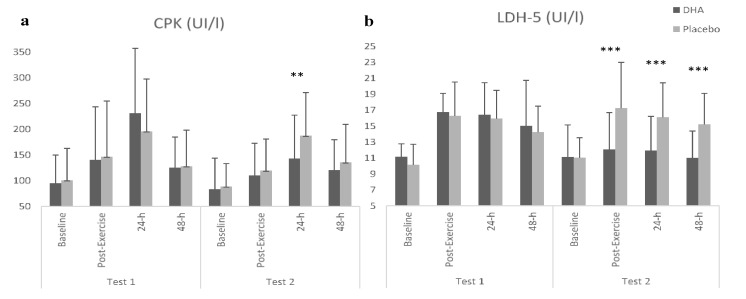
Results of muscle damage markers. **a**: CPK; **b**: LDH-5. ** Differences between DHA + EPA and Placebo *p* < 0.01; *** *p* ≤ 0.001.

Regarding muscle damage markers (Table 2), a significant main effect (supplement × test × time) was observed in the CPK (*F =* 6.251; *p =* 0.014) and in LDH-5 (*F =* 2.713; *p =* 0.05), showing significantly lower values after DHA + EPA supplementation. In this sense, significantly lower values of CPK were observed in the second test after DHA + EPA ingestion at 24 h than at the same point after placebo (*p =* 0.009) (Figure 4a). Likewise, compared with the first testing session, significantly lower values of CPK were shown 24 h after exercise in the second test after DHA + EPA ingestion (*p =* 0.001). In addition, when comparing to placebo, LDH was significantly lower post-exercise (*p =* 0.001), 24 h (*p <* 0.001) and 48 h (*p =* 0.001) after exercise in the second test after DHA + EPA ingestion (Figure 4b). Moreover, compared with the first test, lower values of LDH after DHA + EPA ingestion were shown post-exercise (*p =* 0.001), 24 h (*p <* 0.001) and 48 h (*p =* 0.015) after exercise. However, no effect was observed on LDH-1, LDH-2, LDH-3 and LDH-4.

**Figure 5:**
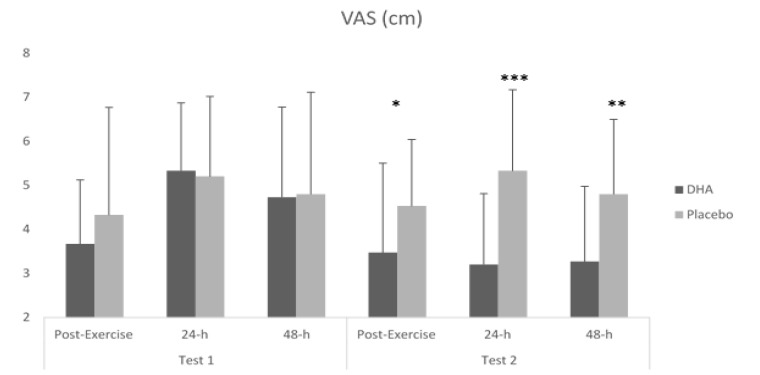
VAS (visual analogy scale) results of muscle damage markers. * Differences between DHA + EPA and Placebo *p* ≤ 0.05; ** *p* ≤ 0.01; *** *p* ≤ 0.001.

With respect to isokinetic results (Table 3), no main effect was found for these variables. Furthermore, no main effect was observed for the rating of perceived exertion. However, concerning muscle soreness, there was a significant effect (supplement × test × time) on the VAS score (*F =* 3.358; *p =* 0.049), showing lower pain values after DHA + EPA consumption. In this way, in comparison with the first test, significantly lower values of VAS after DHA ingestion were shown post-exercise (*p =* 0.001), and 24 h *(p =* 0.023) after exercise. In addition, when compared with the placebo, VAS scores were significantly lower immediately after exercise (*p =* 0.012) and 24 h (*p ≤* 0.001) and 48 h (*p =* 0.002) after exercise in the second test after DHA + EPA ingestion (Figure 5).

## 4. Discussion

To the best of our knowledge, this is the first study to investigate the effects of 10 weeks of docosahexaenoic acid supplementation on strength performance, muscle soreness and inflammatory and muscle damage markers using a randomized, double-blinded cross-over controlled design in trained endurance athletes. We found that ingestion of 2.1 g/day of DHA + 240 mg/day of EPA for 10 weeks did not improve the decreased muscle strength according to baseline values (strength deficit) after an eccentric training session. In addition, DHA + EPA intake did not modify the rating of perceived exertion of the training session. However, significantly lower concentrations of inflammation (IL1β and IL6) and muscle damage markers (CPK and LDH-5) were observed after DHA+EPA supplementation in comparison with the placebo. Moreover, athletes reported lower muscle soreness (measured by VAS) after DHA + EPA supplementation in comparison with a placebo.

Eccentric exercise similar to that performed by the participants of the present study produces muscle myofibril ruptures causing DOMS, inflammation and muscle swelling, limiting the range of movement and decreasing muscle strength [6]. These phenomena have a negative and uncomfortable effect on athletes during the 1–3 days after exercise [26,27] if they want to perform exercise or training. In this way, some previous studies have studied the effect of PUFAs supplementation as an aid to prevent and alleviate muscle damage after eccentric exercise [15,19]. Specifically, Di Lorenzo et al. found an ergogenic effect of 4 weeks of DHA supplementation (2 g/day) on muscle damage after eccentric exercise, showing inhibition of creatine kinase elevation [15]. Our results are in agreement with these previous findings, because we reported significantly lower concentrations of muscle damage markers (CPK and LDH-5) after DHA + EPA supplementation in comparison with a placebo. However, other results are controversial, because another study found no positive effect of 0.8 g/day of DHA supplementation over 2 weeks on muscle damage markers (i.e., creatine kinase) [19]. These divergent results could be related to the dosage of DHA and the duration of supplementation. It seems that ~2 g/day of DHA could prevent muscle damage after eccentric exercise, suggesting less myofibrillar disturbance. In addition, population characteristics can also justify our findings because our study was the first research which used endurance athletes as a sample while most previous studies used untrained subjects [15,19]. Specifically, there was a reduction in the magnitude of expression of markers of muscle damage in the trained population compared with untrained and/or sedentary individuals [28]. Therefore, although muscle damage in this population should be attenuated by their level of training, the supplementation used decreased the expression of markers of muscle damage.

Notably, muscle damage caused by eccentric exercise is caused by micro-damage to muscle fibres, oxidative stress and inflammatory responses [29,30,31]. Therefore, a possible explanation for the protective effect of DHA on muscle damage can be related to the anti-inflammatory effect associated with this fatty acid. In this way, an anti-inflammatory response after 4 weeks of 2 g/day DHA supplementation has been described in the following days after acute eccentric exercise [15]. In addition, Philips et al. [19] found similar results after 2 weeks of 0.8 g/day of DHA supplementation. In agreement with these two previous reports, our results show that 10 weeks of DHA + EPA ingestion promoted lower values of inflammatory markers (statistical significantly in IL1β and a statistical trend in IL6) after exercise (post-exercise) and during the following recovery days (24 h and 48 h). This anti-inflammatory potential of DHA after eccentric exercise is associated with the incorporation of this fatty acid into phospholipidic cell membranes, which decreases the genomic activation of NK-KB cells [1,2] and modifies inflammation mediation processes [3]. In addition, DHA is shown to increase endogenous antioxidant activity via the induction of intracytoplasmic glutathione synthesis [32]. This muted inflammatory response has been previously suggested as a positive effect that moderates the damage response though a DHA intervention, and it may enhance the rate of muscle recovery [33]. Thus, taken together, the current findings and those of previous studies support the idea that DHA supplementation produces an effective ergogenic effect on inflammation after eccentric exercise.

Concerning muscle soreness, controversial findings have been reported on the effects of omega-3 intake on DOMS after eccentric exercise [17,18,34,35]. Many studies have reported a decrement of DOMS after EPA and DHA intake [17,18], but no change was observed after 2–4 weeks of single intake of DHA (0.8–2 g/day) [15,19]. Interestingly, our results are not in accordance with those of previous studies, because in comparison with the placebo, participants showed lower muscle soreness values after DHA consumption immediately after exercise and during the 2 following days (at 24 h and 48 h). To explain these controversial results, a previous review [6] suggested that EPA and DHA supplementation had a certain effect on inhibition of DOMS after eccentric exercise, but the results were related to the omega-3 intake, the dose of fatty acid intake and the muscle groups which performed the exercise. In this way, the previous studies which analysed DHA effect on DOMS used an exercise focused on the elbow flexors [15,19]. However, the exercise-induced DOMS proposed in the present study is focused on the lower limb muscles (half-squats and jumps). Thus, the differences in the muscles involved in the exercise could affect the results. In addition, another factor that may explain these divergent results is the duration of the study. The duration of our supplementation was 10 weeks, much longer than the 2 [19] or 4 weeks [15] used in previous DHA supplementation research. Moreover, training status could affect the effectiveness of DHA supplementation because it has been previously reported that trained individuals can develop more severe soreness with lower muscle damage than untrained individuals [36]. Thus, as we explained above, in our article, the sample consisted of amateur endurance trained athletes in comparison with the untrained subjects of previous studies with DHA [15,19]. According to this finding, one might think that a greater analgesic effect and lower muscle soreness values could be obtained in an untrained sample with the same supplementation treatment. Therefore, to our knowledge, this is the first study which reported that the supplementation of 2 g/day of DHA + 240 mg/day of EPA daily for 10 weeks obtained a reduction in muscle soreness. However, the muscle soreness results of the present study may have been affected by the repeated bout effect. This muscle adaptation promotes a muted damage response after a second eccentric bout by the reparation of the damaged fibers and the incorporation of additional sarcomeres in series [37]. Therefore, during the first 3 or more weeks, the muscles have adapted to the first bout of exercise, providing them protection against further damage [37]. Of note, in our study, there were ten weeks between the first and the second testing sessions after each condition (placebo or DHA) and 4 weeks between the last testing session of one condition and the first training session of the other one (after wash out). Thus, taken together, these findings suggest that this repeated bout effect may been present during the following eccentric training sessions during the present study, and this may have affected the muscle soreness results.

Although omega-3 PUFAs, along with EPA and DHA, have been recently identified as one of the supplementation strategies to restore force in damaged muscles [17,35,38,39], the connection between the inflammatory response and recovery from muscle damage and the attenuation of a strength deficit in muscles caused by eccentric exercise is complex and controversial. In this way, a previous study [15] evaluated the isokinetic strength of elbow flexors after 2 g/day ingestion of DHA over 4 weeks and demonstrated no significant differences in muscle strength reduction but did find an anti-inflammatory response and muscle damage decrement between DHA and placebo in untrained subjects. In agreement with this previous DHA study, we found that the ingestion of 2.1 g/day of DHA + 240 mg/day of EPA for 10 weeks did not improve the strength deficit after an eccentric training session. However, a recent study found that omega-3 fatty acid supplementation (551 mg of EPA and 551 mg of DHA twice daily) for 5 weeks of pre-season training reduced muscle soreness and produced better maintenance of explosive power in professional Rugby Union players [40]. Moreover, an acute dose of 750 mg of EPA + 50 mg of DHA ameliorated the functional changes (jump height) following exercise-induced muscle damage [41]. These controversial findings could be explained by the different strength protocols used to evaluate muscle deficit, the groups of muscles assessed, the differences in eccentric-induced muscle damage exercise or the type of omega-3 supplementation and the dosage intake. In this way, a higher number of eccentric muscle contractions increases muscle damage and produces a slower recovery [28]. The number and the type of eccentric contractions proposed in our study (48 in half-squat and 80 during jumps) are higher and different to those in previous studies (30–60 contractions of elbow flexors) [15,19]. This fact may also modify the controversial findings about the effectiveness of DHA + EPA against a strength deficit.

In addition, DHA supplements in the market may consist of ethyl esters, free fatty acids, monoacylglycerols or regular triglycerides. This disparity in supplement composition may have contributed to the controversial findings of previous studies. In this way, the supplement used in the present study (Tridocosahexaenoine-AOX^®^) has a different composition from other fish oils previously used in sport supplementation. Specifically, the product used in the present study is obtained by enzymatic synthesis from tuna fish oil [42] and composed of almost only DHA in triglyceride form (representing 70% of total fatty acids and 90% of total omega-3 PUFAs) after re-esterification, with a high proportion in the second glycerol position (sn-2). On the other hand, natural fish oils use to contain low amounts of DHA. However, in re-esterified triacyclglycerol formulas, a random re-esterification can place another DHA molecule in the sn-1, sn-3 or both positions, hence the high proportion of DHA in the final formula [43]. Furthermore, the DHA supplement used in the present study has previously demonstrated, in in vitro [30,39] studies, a great antioxidant and anti-inflammatory activity. Thus, this configuration can be more favourable and likely have a positive influence on bioavailability [44] that could justify the results of the present study.

The main limitation of the present study was the small number of subjects enrolled and the consequent low statistical significance of the results. From an applied perspective, physicians, nutritionists, coaches and athletes should bear in mind that if the exercise performed can generate DOMS (e.g., unaccustomed exercises, high-intensities, ultra-endurance races etc.) the intake of DHA + EPA supplementation (2.1 g/day + 240 mg/day respectively) is a suitable way to minimize inflammatory and muscle damage responses and muscle soreness perception of the athletes. In addition, the DHA + EPA supplementation in sport modalities which need to develop simultaneously several physical capacities or need to train 2–3 times per day should be an interesting aid due to its faster time course of recovery. In addition, it is necessary to analyse the effect of this type of supplementation on strength deficit using other strength test (e.g., 1-RM) because the stress of the eccentric-induced muscle damage task selected in the present study was half-squat, and it may be more specific to tested by a 1-RM in half-squat. This fact may also be considered a potential limitation. Therefore, this research unveiled a new line of supplementation with re-esterified DHA + EPA aimed at maximizing recovery and performance after an eccentric exercise in endurance sports.

## 5. Conclusions

Ten weeks of re-esterified docosahexaenoic acid supplementation (2.1 g/day) and eicosapentaenoic acid (240 mg/day) promoted lower concentrations of inflammation (IL1β and IL6) and muscle damage markers (CPK and LDH-5) and decreased muscle soreness but did not improve the knee flexors and extensor strength deficit after an eccentric training session in trained endurance athletes.

## Figures and Tables

**Table 1:** Results of inflammatory markers in each testing session and time after placebo or DHA ingestion.

Outcome	Testing Session	Baseline		Post-exercise		post 24 h		post 48 h		ANOVA (supplement × test ×time)		Post-hoc	
		Mean	SD	Mean	SD	Mean	SD	Mean	SD	*F*	*p*	Comparison	*p*
IL1β (pg/ml)	Pre-DHA + EPA	2.18	1.29	2.80	1.21	2.78	1.49	2.64	1.59	4.247	0.011	DHA + EPA test 1 vs. DHA + EPA test 2 (post-exerc)	**0.036**
	Post-DHA + EPA	2.22	1.44	2.49	1.42	2.48	1.48	2.24	1.40			DHA + EPA test 1 vs. DHA + EPA test 2 (24 h)	**0.002**
	Pre-Placebo	2.17	1.12	2.73	1.12	2.73	1.29	2.54	1.36			DHA + EPA test 1 vs. DHA + EPA test 2 (48 h)	**0.004**
	Post-Placebo	1.94	1.17	2.90	1.78	2.86	1.50	2.35	1.28				
IL6 (pg/ml)	Pre-DHA + EPA	2.55	1.29	3.53	1.45	3.12	1.50	2.74	1.55	5.909	0.009	DHA + EPA test 1 vs. DHA + EPA test 2 (post-exerc)	0.062
	Post-DHA + EPA	2.75	1.70	3.07	1.77	2.80	1.76	2.98	2.00			DHA + EPA test 1 vs. DHA + EPA test 2 (24 h)	0.067
	Pre-Placebo	3.02	1.71	3.95	1.89	3.64	1.97	3.07	1.66				
	Post-Placebo	2.66	1.71	3.89	2.32	3.51	2.18	3.16	1.94				
IL8 (pg/ml)	Pre-DHA + EPA	4.04	0.81	5.12	1.03	4.40	1.06	4.40	1.25	1.728	0.197		
	Post-DHA + EPA	4.09	1.01	4.70	1.00	4.41	1.08	4.15	1.10				
	Pre-Placebo	3.86	1.00	4.80	1.12	4.30	1.31	4.14	1.40				
	Post-Placebo	4.01	1.33	5.25	1.43	4.76	1.56	4.29	1.46				
C-RP (mg/dl)	Pre-DHA + EPA	0.97	0.89	0.95	0.78	1.12	1.17	1.08	1.13	0.041	0.920		
	Post-DHA + EPA	1.20	0.89	1.14	0.84	1.13	0.75	0.77	0.59				
	Pre-Placebo	0.80	0.80	0.72	0.79	1.01	1.39	0.94	1.37				
	Post-Placebo	1.23	1.35	1.18	1.36	1.28	1.62	0.99	1.25				
TNFα (pg/ml)	Pre-DHA + EPA	7.77	1.02	9.18	1.24	9.10	1.22	8.65	1.79	1.228	0.290		
	Post-DHA + EPA	8.36	1.38	9.16	1.56	9.05	1.81	8.95	2.12				
	Pre-Placebo	8.22	1.53	9.36	2.35	9.36	1.80	8.69	2.06				
	Post-Placebo	7.77	1.06	9.11	1.11	9.46	2.16	8.52	1.47				

C-RP: C-reactive protein; IL: Interleukin; TNFα: Tumor necrosis factor α.

**Table 2:** Results of muscle damage markers in each testing session and time after placebo or DHA ingestion.

Outcome	Testing Session	Baseline		Post-exercise		post 24 h		post 48 h		ANOVA (supplement × test × time)		Post-hoc (Bonferroni)	
		Mean	SD	Mean	SD	Mean	SD	Mean	SD	*F*	*p*	Comparison	*p*
CPK (UI/l)	Pre-DHA + EPA	94.54	55.24	140.23	103.17	230.62	126.18	124.92	59.78	6.251	0.014	DHA + EPA test 1 vs. DHA + EPA test 2 (24 h)	**0.001**
	Post-DHA + EPA	83.15	60.32	109.77	62.53	142.69	84.51	120.31	59.08				
	Pre-Placebo	100.38	62.34	146.23	108.29	195.54	101.65	127.77	70.22				
	Post-Placebo	88.23	44.79	119.23	61.39	187.00	84.14	135.00	74.17				
LDH-1 (UI/l)	Pre-DHA + EPA	26.99	4.83	25.33	4.25	27.46	4.96	28.28	5.05	1.251	0.307		
	Post-DHA + EPA	27.17	5.45	27.26	5.53	27.60	4.60	28.89	3.95				
	Pre-Placebo	29.61	4.87	27.20	5.96	28.56	4.87	30.64	3.86				
	Post-Placebo	26.83	3.63	25.01	2.91	28.53	2.65	29.10	3.71				
LDH-2 (UI/l)	Pre-DHA + EPA	33.98	2.63	30.18	3.53	32.03	3.07	33.61	4.30	1.483	0.252		
	Post-DHA + EPA	31.78	3.99	31.18	3.87	30.33	3.49	31.24	3.03				
	Pre-Placebo	33.62	2.82	31.93	3.36	32.78	2.75	35.54	2.84				
	Post-Placebo	30.43	4.15	28.86	2.97	30.91	2.40	31.50	2.57				
LDH-3 (UI/l)	Pre-DHA + EPA	19.44	2.53	18.15	2.35	18.61	1.98	17.55	1.63	1.410	0.260		
	Post-DHA + EPA	19.55	2.08	19.13	2.54	18.89	1.71	18.70	2.05				
	Pre-Placebo	18.33	3.80	18.41	3.31	18.80	3.02	17.79	2.21				
	Post-Placebo	19.62	1.85	18.82	1.84	18.66	1.48	17.92	2.47				
LDH-4 (UI/l)	Pre-DHA	8.21	1.69	9.59	2.37	8.27	2.18	7.28	2.17	0.733	0.540		
	Post-DHA	8.08	2.27	8.53	2.00	8.80	2.04	8.18	1.43				
	Pre-Placebo	7.97	2.40	8.81	2.90	7.84	2.44	5.80	1.79				
	Post-Placebo	9.03	2.02	9.60	1.38	8.79	1.05	7.81	1.44				
LDH-5 (UI/l)	Pre-DHA + EPA	11.15	1.62	16.73	2.37	16.42	4.01	15.00	5.72	2.713	0.05	DHA + EPA test 1 vs. DHA + EPA test 2 (post-exerc)	**0.001**
	Post-DHA + EPA	11.11	4.03	12.06	4.62	11.93	4.28	11.00	3.37			DHA + EPA test 1 vs. DHA + EPA test 2 (24 h)	**<0.001**
	Pre-Placebo	10.13	2.58	16.27	4.24	15.94	3.53	14.23	3.26			DHA + EPA test 1 vs. DHA + EPA test 2 (48 h)	**0.015**
	Post-Placebo	11.03	2.51	17.23	5.76	16.09	4.32	15.21	3.89				

CPK: creatine phosphokinase; LDH: Lactate dehydrogenase.

**Table 3:** Results of isokinetic performance, pain and rating of perceived exertion in each testing session and time after placebo or DHA ingestion.

Outcome	Testing Session	Baseline		Post-exercise		post 24 h		post 48 h		ANOVA (supplement × test × time)		Post-hoc (Bonferroni)	
		Mean	SD	Mean	SD	Mean	SD	Mean	SD	*F*	*p*	Comparison	*p*
VAS (cm)	Pre-DHA + EPA			3.67	1.45	5.33	1.54	4.73	2.05	3.358	0.049	DHA + EPA test 1 vs. DHA + EPA test 2 (24 h)	**0.001**
	Post-DHA + EPA			3.47	2.03	3.20	1.61	3.27	1.71			DHA + EPA test 1 vs. DHA + EPA test 2 (48 h)	**0.023**
	Pre-Placebo			4.33	2.44	5.20	1.82	4.80	2.31				
	Post-Placebo			4.53	1.51	5.33	1.84	4.80	1.70				
RPE (AU)	Pre-DHA + EPA			17.57	2.06					0.741	0.638		
	Post-DHA + EPA			17.79	1.63								
	Pre-Placebo			17.57	1.65								
	Post-Placebo			17.71	2.09								
Peak Torque Flexion (N·m^2^)	Pre-DHA + EPA	95.59	12.02	90.01	15.46	93.64	16.83	94.64	14.86	0.050	0.985		
	Post-DHA + EPA	94.84	14.50	89.76	18.48	89.61	16.81	88.44	17.45				
	Pre-Placebo	97.93	17.66	91.11	15.37	93.06	16.15	91.81	16.16				
	Post-Placebo	93.36	14.77	87.65	13.81	86.24	15.60	83.22	15.16				
Peak Torque Extension (N·m^2^)	Pre-DHA + EPA	173.93	23.04	156.44	23.93	162.79	28.18	166.50	27.29	0.364	0.780		
	Post-DHA + EPA	176.67	25.53	159.25	28.35	161.47	27.28	160.45	28.87				
	Pre-Placebo	175.68	28.04	158.27	24.04	163.81	23.26	166.63	26.58				
	Post-Placebo	176.25	20.15	151.84	24.16	158.94	27.48	156.16	29.43				

VAS: visual-analogy scale; RPE: rating of perceived exertion.
